# Macromolecular Condensates as Tunable Scaffolds for Bio‐Inspired Silica Hybrids

**DOI:** 10.1002/anie.202522966

**Published:** 2026-02-17

**Authors:** Protap Biswas, Lior Aram, Nitzan Livni, Roman Kamyshinsky, Nadav Elad, Michal Leskes, Assaf Gal

**Affiliations:** ^1^ Department of Plant and Environmental Sciences Weizmann Institute of Science Rehovot Israel; ^2^ Department of Molecular Chemistry and Materials Science Weizmann Institute of Science Rehovot Israel; ^3^ Department of Chemical Research Support Weizmann Institute of Science Rehovot Israel

**Keywords:** Bio‐silicification, CryoEM, liquid–liquid phase separation, macromolecular condensates, nanoparticle synthesis

## Abstract

Silica‐based materials are of immense functionality as their production is versatile and can accommodate a wide range of properties. Nevertheless, no synthetic system can reproduce the ability of organisms to precipitate dense silica under ambient conditions and from dilute soluble precursors, leaving a substantial gap in our understanding of silica chemistry. It is widely accepted that a key feature of biosilicification is the activity of amine‐rich macromolecules, but their biomimetic use in silica synthesis currently fails to reproduce biological processes. Here, we take inspiration from some properties of biological processes and demonstrate that phase separated polyamine condensates drive the formation of hybrid silica materials. We further show that the pH of the reaction is a regulator that allows to control the architecture and composition of the silica material. These results point to the fundamental role of condensates in driving silicification from dilute aqueous environments that characterize physiological conditions. Applying these sets of rules to synthetic systems may open the road for the production of a new class of dense and biocompatible silica hybrids.

## Graphical abstract

Dense bio‐inspired silica formation is achieved by coupling polyamine liquid–liquid phase separation with pH‐dependent silica polymerization. Silicification within polyamine condensates, enables precise control over particle size, density, and morphology. Mild basic aqueous conditions yield silica hybrids approaching the density and low porosity of biologically formed silica, with pH directing transitions from uniform to hollow silica structures.

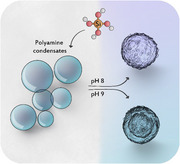

## 1 Introduction

Silica is a highly versatile material with various applications, varying in their requirements for chemical, mechanical, physical, and optical properties [1, 2, 3, 4, 5]. Historically, high‐temperature manufacturing was mandatory in the processing of this inorganic polymeric substance [6]. However, the development of sol‐gel routes allowed the production of new generations of materials that form under mild, biocompatible conditions [7, 8, 9]. The distinctive aqueous environment of sol‐gel chemistry results in diverse porous materials that are beneficial for applications such as catalysis and separation. Nevertheless, in other cases, a space‐filling material is preferred, but the uncontrolled gelation process limits the ability to form compact structures and to tailor their architecture. As a result, producing dense silica nanoparticles usually requires the presence of structure‐directing agents and much harsher chemical conditions [5].

In contrast, living organisms such as the silicifying algae diatoms, form dense biosilica at ambient conditions, from soluble building blocks [10]. This biosilica is characterized by intricate architectures and superb mechanical properties [11]. The physiological process of biosilica formation serves as an inspiration source for scientific attempts to improve control over silica‐based materials [2, 3, 12, 13]. A key feature is the fact that diatom silica is an organic‐inorganic hybrid that is characterized by the presence of amine‐rich macromolecules [14, 15, 16, 17, 18]. Many efforts were invested in understanding how such polyamines can direct the process of silica polymerization, nevertheless, a basic chemical reasoning is still incomplete [19, 20, 21, 22, 23, 24, 25, 26, 27]. The common approach was to include the polyamines as soluble additives in the sol‐gel process and to tune their interactions with the silicate species. However, the results varied greatly in the properties of the forming materials, depending on the exact chemistry of the experimental system.

A less‐studied mechanism that can affect silica polymerization is liquid–liquid phase separation (LLPS) [28, 29]. LLPS in the context of biosilicification was discussed decades ago, given the electrostatic forces that persist between charged silicates and polyamines at different pH values. However, because in most cases the investigations were carried out on reactions at highly metastable conditions, when silica is supersaturated and kinetic considerations dominate, the involvement of a LLPS process was described only in a qualitative, experiment‐specific manner [20, 24, 26, 29, 30, 31, 32, 33, 34, 35, 36, 37, 38]. These limitations were partially addressed in a recent work, where we have shown that mixing two dilute solutions of polyamines and silicates induces a mutual phase separation into liquid precursors in a pH‐dependent manner from which silica polymerizes [39]. However, in contrast to biosilicification, this silicate‐polymer phase separation yields a hybrid material with a random network architecture rather than a densely packed material.

A notable difference between the polyamine‐silicate synthetic systems and biosilicification is that the chemical environment inside cells is crowded with macromolecules and very different from dilute solutions with soluble solutes, such as the ones used previously [39]. In the last two decades, the concept of macromolecular condensates that form via a LLPS process is revolutionizing cell biology and materials science alike [40, 41, 42, 43, 44, 45]. These dense polymer droplets are characterized by a distinct chemical environment that is different from the surrounding dilute phase, leading to emergent chemical properties such as accelerated kinetics and interfacial gradients. It is now widely realized that the outcome of chemical reactions in phase separated systems is fundamentally different from a single dilute phase and from other colloidal systems [46, 47, 48].

In this study, we investigate the hypothesis that silicate‐polyamine interactions within dense polyamine condensates, rather than in soluble conditions, will yield dense silica particles. The results show that combining polyamine LLPS process with the pH dependent speciation of silicate species can be used to fine‐tune the formation of silica particles in mild aqueous conditions, resulting in silica hybrids that are made of silica that is almost as non‐porous and dense as biologically formed silica. The initial formation of polymer droplets serves as a first step to control size and composition, whereas the subsequent silicification step controls the final architecture from space‐filling to hollow particles, depending on the pH of the silicate precursor. The observation that dense silica formation is preferred at mildly basic conditions in macromolecular condensates might be the key to developing soft chemistry silica manufacturing under physiological conditions.

## 2 Results

**FIGURE 1:**
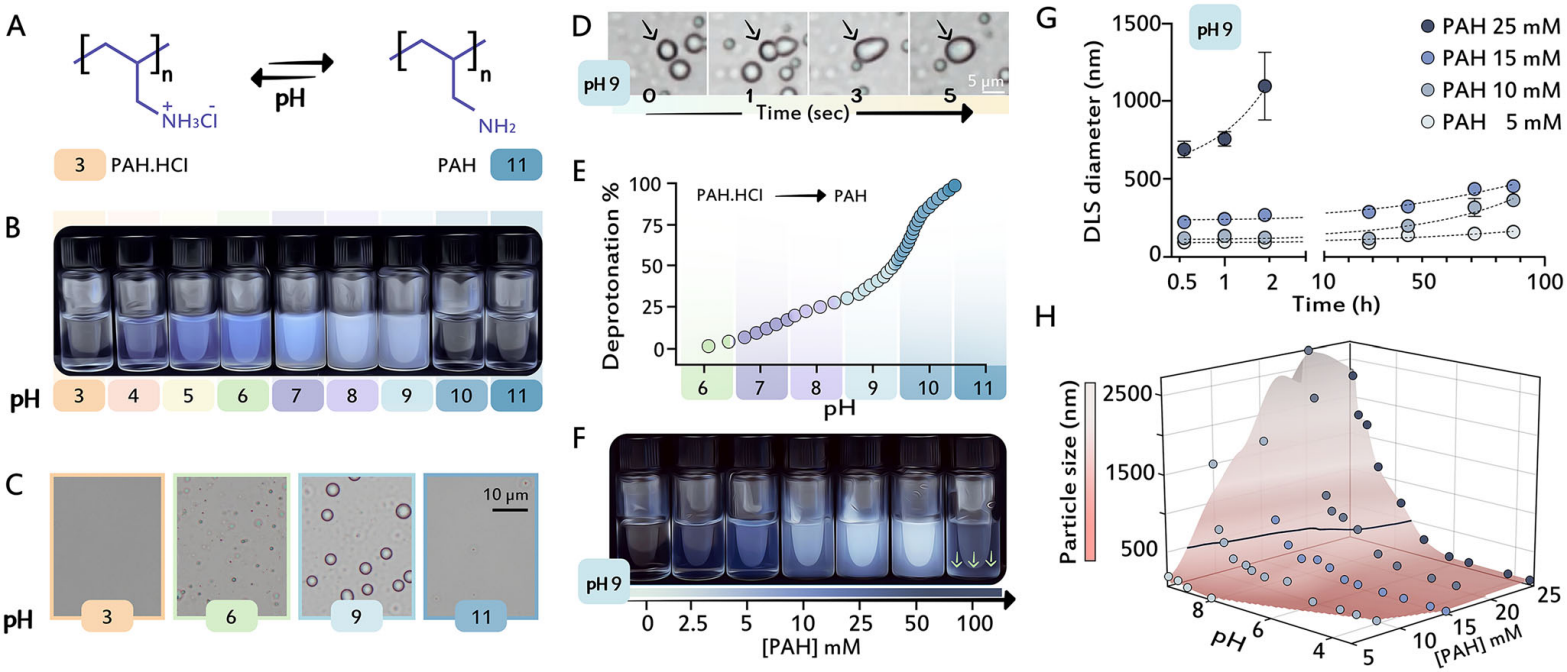
LLPS of PAH is pH and concentration dependent. (A) Chemical structure of protonated and deprotonated states of polyallylamine. (B) Solutions of 25 mM PAH are showing increased turbidity with increasing pH at the pH range of 4 to 9. (C) Optical microscopy images of PAH solutions at different pH conditions. Increased turbidity from pH 6 to 9 emerges from the presence of larger droplets. (D) Snapshots of 25 mM PAH at pH 9 demonstrating the coalescence (arrowheads) of liquid PAH droplets into bigger droplets. (E) A deprotonation curve of 25 mM PAH as a function of pH. See also Figure S2. (F) Turbidity of PAH solutions at different concentrations, all at pH 9. Yellow arrows at 100 mM indicate a macroscopic boundary between dilute and dense phases. (G) DLS measurements of droplet size show a time‐dependent coalescence that is slowing down for lower concentrations. (H) Surface plot depicting particle size distribution measured by DLS as a function of PAH concentration and pH. The black line is a 500 nm contour showing the various combinations of pH and concentration that yield similar particle size.

As a first step, we aimed to establish a synthetic macromolecular condensate system, where polyamines phase‐separate into liquid condensates before the introduction of the silicates. To establish such a system, we studied a 50 kDa polyallylamine (PAH) hydrochloride. This polymer has a higher molecular weight than the previously used PAH [39]. This length seems to be close to the minimal solubility of PAH, as longer polymers are again miscible at comparable concentrations (Figure S1). This reduced miscibility makes it more liable to phase separation, leading to preferential liquid droplet formation. As polyallylamine is a weak organic base sensitive to pH (Figure 1A), we initially investigated its behavior over various pH values. At acidic conditions, 25 mM PAH is soluble, but above pH 4 the solution evolves turbidity that increases until pH 9 (Figure 1B). Examining the turbid PAH solutions with optical microscopy showed that they consist of spherical droplets that aggregate with time, as expected from phase separated liquid droplets (Figure 1C,D). Above pH 9, the fraction of neutral amine residues becomes dominant (Figure 1E), and PAH turns miscible again (Figure 1B,C), probably due to charge compensation effects. These observations demonstrate a pH‐controlled LLPS behavior of the PAH molecules.

Apart from pH, also polymer concentration should affect the process of phase separation. Indeed, when PAH concentrations were elevated at pH 9, turbidity increased as well, until at 100 mM PAH, macroscopic phase separation was observed (Figure 1F). Even though at the higher polymer concentrations the droplets coalesce and grow with time, we note a non‐equilibrium aging effect at lower concentrations [49], as coalescence slowed down with time (Figure 1G). Finally, in order to quantify the dual effect of pH and PAH concentrations, we systematically investigated with DLS the sizes of droplets that formed directly after LLPS in this two‐dimensional parameter space (Figure 1H). This shows a combinatorial effect on droplet size, where pH causes exponential increase, and concentration has a linear effect. Altogether, we have established a PAH‐based system that undergoes LLPS and can be used to control the size of dense PAH droplets by tuning the concentration of the polymer, solution pH, and the time for droplet coalescence.

**FIGURE 2:**
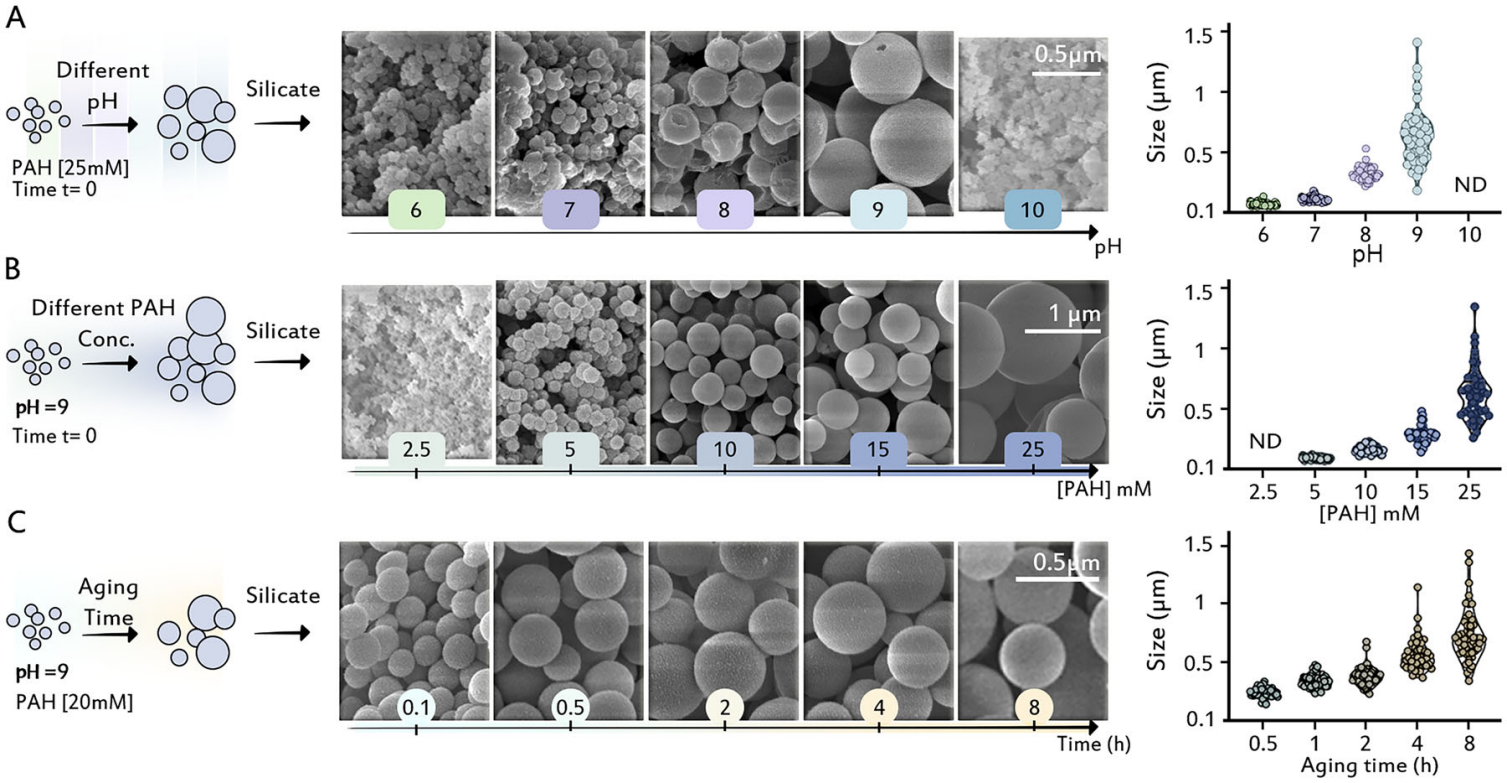
Silicification of PAH condensates yield solid particles with controlled sizes. (A) SEM images of pH‐dependent silicification of PAH droplets. Silicate was added immediately after PAH LLPS and the post‐silicification time was 4 h to prevent gelation in the dilute phase below pH 8. Note that at pH 10, a connected network is observed, which is characteristic to a single‐phase system. (B) Concentration‐dependent silicification of PAH droplets. (C) PAH droplets were aged before silicification for the indicated times. This caused droplet coalescence and larger sizes. Statistical analyses of particle diameter are presented as violin plots (*n* = 50) for each experimental condition (ND: not determined).

The next step of the experiments was to introduce a soluble silicate source to the phase separated PAH solutions. A 50 mM sodium silicate solution was chosen since its interactions with PAH in a dilute regime were recently studied [39], and it is more similar to environmental silicate sources than organic alkoxy silanes. The silicate solutions were freshly prepared, and their pH was adjusted to be the same as the phase separated PAH solution. Since it was recently shown that PAH can induce silicification even from undersaturated silicate solutions [39], we explored a wide pH range where at the lower end, around pH 6, silica is metastable and spontaneously forms gel, but in the upper pH values, above 8, the silicate solution is undersaturated and stable. Remarkably, upon mixing the silicate solutions and the phase separated PAH solutions (between pH 6 and 9), the dense polymer droplets rapidly solidified. This is deduced from the fact that spherical particles with a similar size distribution to the parent droplets could be isolated from the reaction (Figure 2A), whereas in the absence of silicate, the PAH droplets collapsed upon drying (Figure S3).

It is interesting to compare the dilute phase of this system to the shorter PAH (17.5 kDa) system that consists of a single dilute phase at the entire pH range (Figure S4). Here, we did not observe in the dilute phase the hybrid networks that characterize the silicate‐PAH condensation in the shorter PAH system [39]. This is probably due to the reduced concentration of the polymer after LLPS, which was also manifested in the formation of silica gel in the dilute phase at pH conditions of 6–7. Nevertheless, at pH 10, when also this longer PAH is soluble and does not phase separate, the formation of silica networks was observed (Figure 2A), similar to the 17.5 kDa PAH. This suggests that the mechanism of a PAH‐induced silica polymerization that dominates in single‐phase systems, is suppressed by a more dominant silicification process inside condensates of phase separated systems.

Since silicification is preferred within the condensates, and condensate size is a function of pH, the system offers a tunable experimental handle (the pH value) to control particle size (Figure 2A). We tested if it is possible to control the size of the silica particles with the two other determinants of the LLPS process, PAH concentration and coalescence time. Indeed, particle size was found to increase with increasing concentrations above the critical LLPS concentration (a dilute solution of 2.5 mM shows only the expected silica networks). Similarly, aging of the droplets before the addition of a silicate source causes the resulting particle size to increase in accordance with the expected Oswald ripening (Figures 2B,C and S5). Overall, the PAH droplets proved to be a high‐fidelity reaction hotspot for the formation of solid silica particles from soluble silicate solutions, and their formation inhibited the formation of silica networks that prevailed in miscible PAH conditions.

**FIGURE 3:**
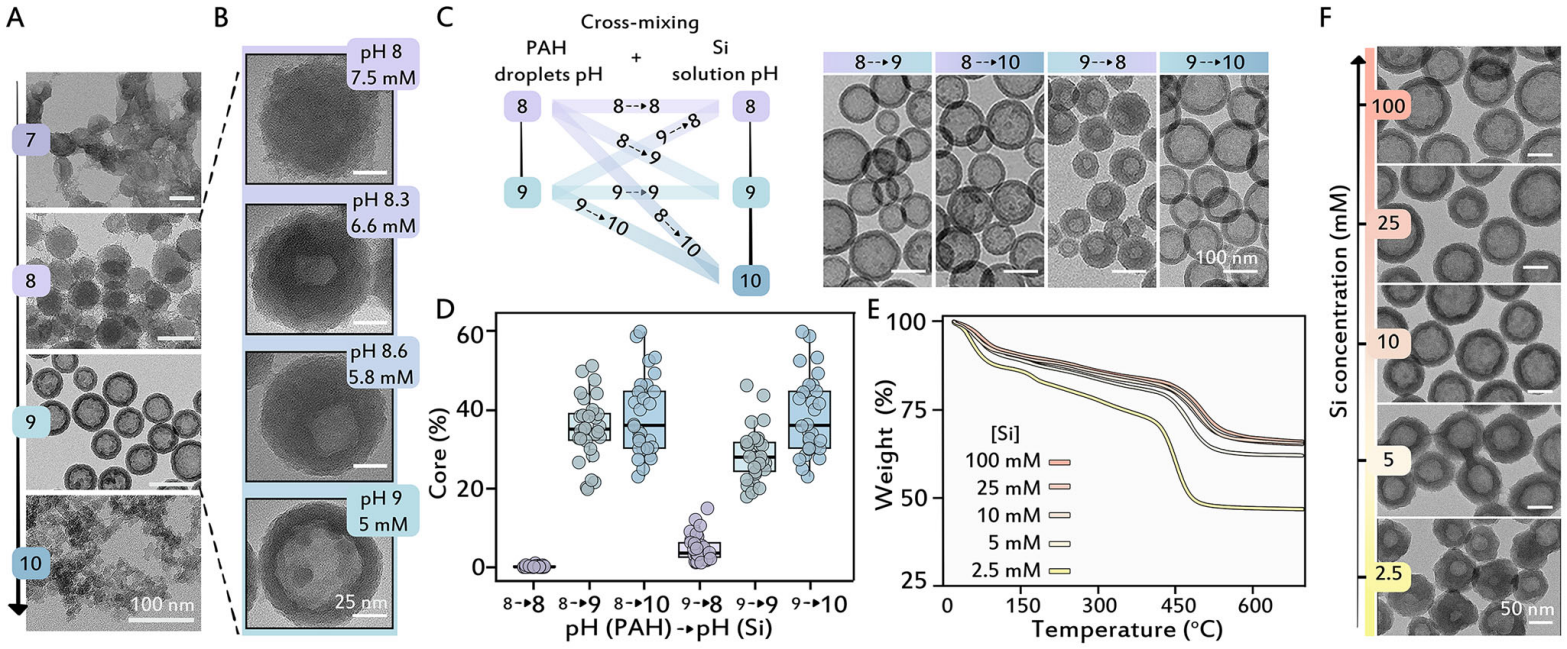
Effects of pH and [Si] on the morphology of silica particles. (A) TEM images of silica particles synthesized at different pH conditions. (B) Morphological comparison at pH range of 8 to 9 of similarly sized silica particles obtained by varying PAH concentration and pH. (C) Silica particles were synthesized by mixing polymer droplets and silicate solutions with different pH values; each pair of numbers, X→Y, indicates the pH of the PAH droplet (X) and the pH of the silicate solution (Y). TEM images of the four combinations of different pH values are shown. (D) Boxplots show the volume fraction of hollow cores in particles obtained from pH cross‐mixing experiments based on TEM image analysis (*n* = 30 each), boxes indicate the interquartile range (IQR) with the median as a horizontal line; whiskers extend to 1.5 × IQR. (E) TGA traces of silica particles synthesized at pH 9 from different silicate concentrations. (F) TEM images of silica particles formed at varying silicate concentrations at pH 9.

We noted that different combinations of the two chemical handles for particle size control, pH and PAH concentration, allow to achieve similar sizes at different conditions (i.e., pH 8 and 7.5 mM PAH gives similar sized particles to pH 9 and 5 mM PAH, see also Figures 1H and S6). This opens the opportunity to investigate if pH conditions have additional effects, other than size regulation. Indeed, imaging similarly sized particles that formed at different pH values with transmission electron microscopy (TEM), revealed a significant morphological difference (Figure 3A). The space‐filling silica particles that formed at pH 8 and lower, gave way to a hollow sphere morphology at pH 9, before turning into the silica networks of the single‐phase at pH 10. Focusing on the pH range of 8 to 9 with better pH resolution, showed a clear pH‐dependent trend where the volume fraction of the hollow core increases with rising pH (Figures 3B and S6). Notably, the formation of the core cavity occurs at all PAH concentrations and is independent of particle size (Figure S7).

pH is a driver of both steps in the process—first, LLPS of the polymer solution, and second, silicification after the addition of the silicate source. To differentiate which step is responsible for the hollow sphere morphology, we mixed PAH solutions that phase separated at one pH with silicate solutions at a different pH value (Figure 3C). These samples were imaged with TEM and the relative size of the hollow cores was quantified using image analysis (Figure 3D). Comparing the volumes of the cores showed a clear correlation between higher pH of the silicate solution and larger cores, which means that silicification pH is the determinant for increased core volume, while the pH of PAH LLPS has a minor effect.

Because diffusion gradients underlie the transport of silicate species into the dense polymer droplets, we tested the effect of different silicate concentrations at pH 9. Thermogravimetric analysis (TGA) showed a trend of lower polymer content in the particles formed at higher Si concentrations, that plateaued at 16%–18% weight fraction of PAH around 25 mM silicate (Figure 3E), and TEM imaging showed similar morphologies at different Si concentrations (Figure 3F). Overall, from all the chemical parameters that were tested, pH of the silicate solution emerges as the dominant factor that is responsible for the architectural switch from space‐filling to hollow spheres.

**FIGURE 4:**
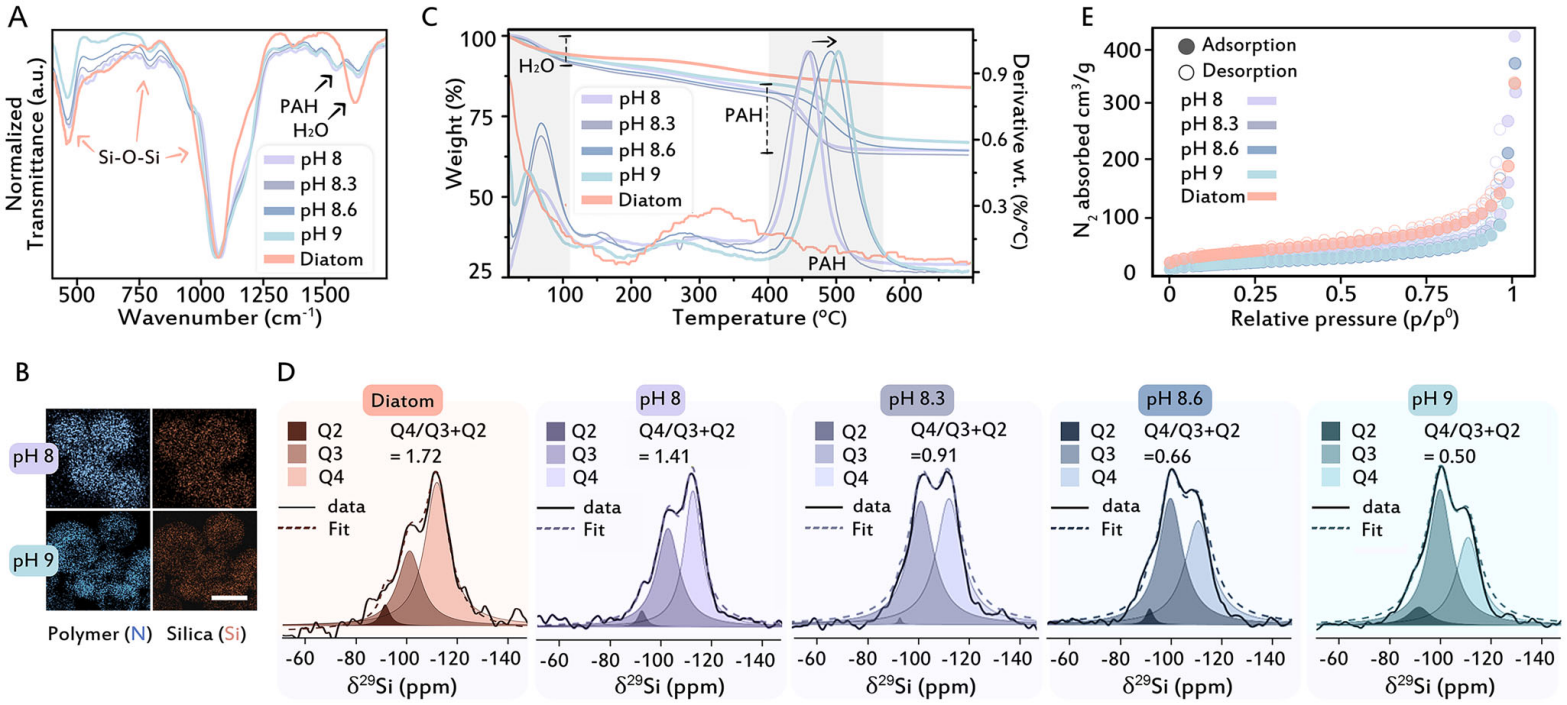
Physicochemical characterization of dried silica particles under different pH conditions and comparison with diatom silica. (A) FT‐IR spectra with characteristic peaks of amorphous silica, occluded water, and PAH. (B) TEM‐EDS mapping in silica hybrids showing the colocalization of N from PAH with Si (scale bar is 100 nm). (C) TGA traces (weight loss on the left Y axis) and their derivatives (right Y axis), of the silica particles, the shaded parts represent the weight losses due to occluded water and polymer degradation. (D) Solid state ^29^Si NMR spectra with increasing pH values (left to right) alongside fitting results for the Q2‐4 peak ratios. (E) Gas adsorption measurement by BET isotherm of the particles shows microporosity indicating nonporous silica formation. See also Table S1.

We performed several physicochemical analyses to characterize dried silica samples that formed between pH 8 and 9, and to compare them to the properties of biological silica extracted from diatoms. Fourier transformed infrared (FTIR) spectroscopy (Figure 4A), and energy‐dispersive x‐ray spectroscopy (EDS) (Figure 4B), confirmed that all samples consisted of hybrids of silica and polymers. TGA revealed nearly similar silica content (64%–67%) in all synthetic samples, with comparable polymer fractions (10%–14%). However, the polymer degradation temperature differed, with particles formed at pH 8 decomposing at ∼460°C, while those formed at pH 9 burned at ∼500°C (Figure 4C). This suggests stronger polymer‐silica interactions at higher pH. Natural diatom silica shows higher silica content (∼82%) and volatile components that decomposed at around 320°C. A similar trend was detected with ^29^Si solid state nuclear magnetic resonance (^29^Si NMR) spectroscopy, where the samples that formed at higher pH had a higher fraction of uncondensed silanol groups (Figures 4D and S8), putatively because of interactions with the polymer residues. We measured BET nitrogen gas adsorption isotherms to quantify the porosity of the silica samples. The BET isotherms show similar surface areas for all samples of about 100 cm^3^/g (Figure 4E and Table S1) and display characteristics of typical nonporous/macroporous materials. Comparing the synthetic samples to diatom biosilica shows that the biogenic silica has sharp FTIR peaks, high degree of silica condensation in NMR, and a low macromolecular content in TGA, with comparable BET surface area and non‐porosity (Figure 4). These properties make the compositional properties of diatom silica more similar to the synthetic samples formed at the lower, and more physiological, pH values, and open the question of what is the mechanism that gives rise to the formation of the hollow core at the higher pH values.

**FIGURE 5:**
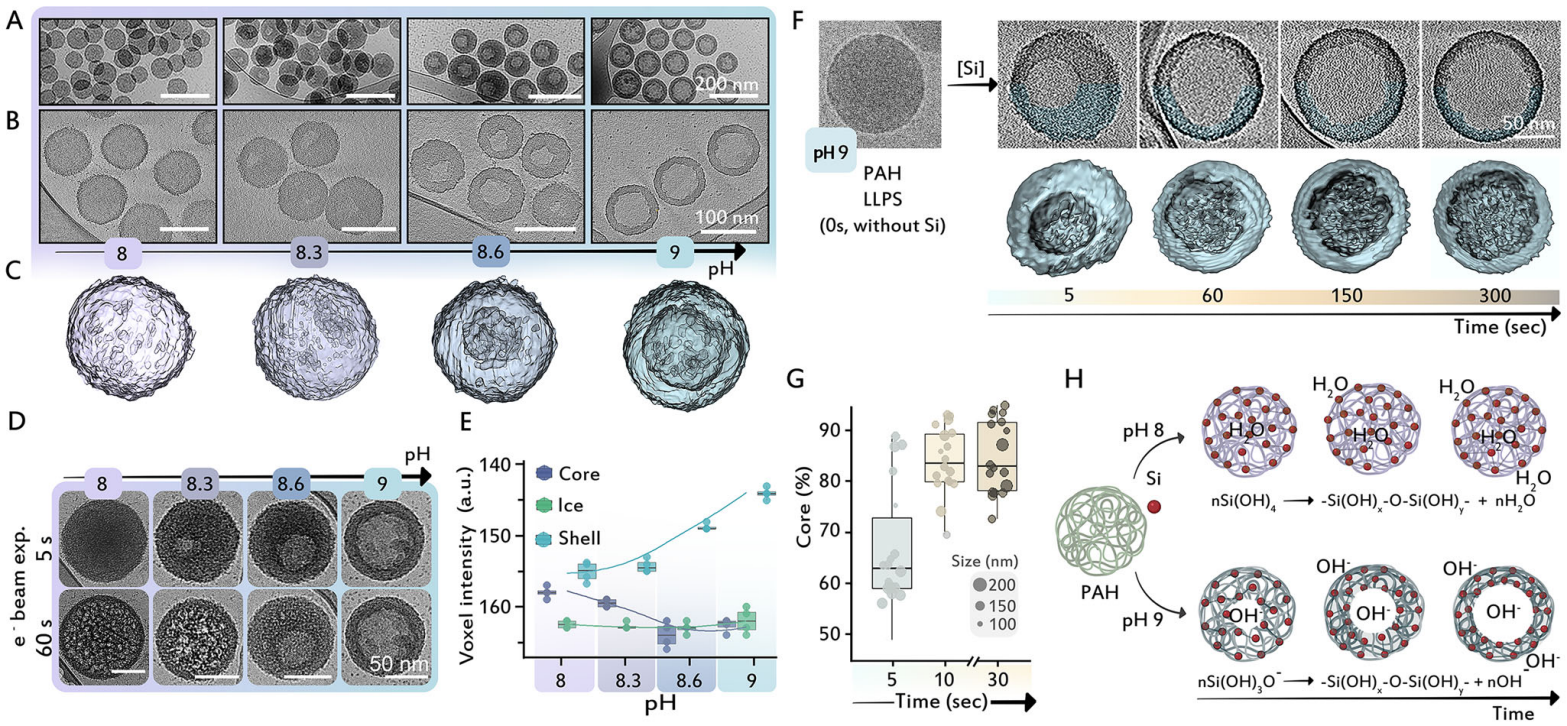
CryoET imaging and analysis of silica particles under varying pH conditions and 50 mM [Si]. (A) Low magnification cryoTEM images of vitrified silica particles formed at various pH values. (B) Corresponding reconstructed tomographic slices from cryoET datasets of the same samples as in (A). (C) 3D volume rendering of representative silicified particles. (D) Cryo‐TEM images of particles exposed to the electron beam (dose rate: 5.61 e^−^/Å^2^) show pronounced damage after 60 s to the particles formed at lower pH. (E) Voxel intensity analysis from tomographic datasets from different locations in the particles. Boxplots show median and IQR of voxel intensity; points indicate individual sample means. (F) Time‐resolved cryoET shows cavity formation dynamics in silica particles at pH 9. (G) Quantification of core fraction over time derived from cryo‐TEM data, boxes indicate 1.5 × IQR, lines show the mean, and point size corresponds to particle size synthesized at pH 9. (H) Schematic representation of silicification pathways at different pH values: uniform particle formation at pH 8 versus cavity formation at pH 9. At elevated pH, the prevalence of silicate anions (Si(OH)_3_O^−^) promotes densification and internal cavity development through templating of Si‐PAH hybrids; ‘x’ and 'y denote an arbitrary number of silanol groups in the condensation reaction.

Since all previous analyses were done on dried samples, we wanted to investigate the properties of the silica particles in their original hydrated condition during the formation process. We vitrified solution aliquots with mature particles and collected 3D cryo electron tomography (cryoET) data that uses the contrast differences between the particles and their surrounding vitrified solution. Qualitatively, cryoET recapitulates the pH‐dependent architecture that was observed in dried samples (Figure 5A–C), where the increasing volume of the core is evident in both 2D images and 3D reconstructions. Additionally, the cryoTEM imaging provided indirect proof for the tighter interactions between PAH and silica at the higher pH values as a much better resistance to beam damage was observed in the particles that formed under higher pH values (Figure 5D).

To gain information on the compositions of the various particles we used the fact that voxel intensity in the cryoET datasets is related to the electron density of the underlying material. Comparing voxel intensities of the particle cores and shells shows a clear trend of increase in the shell voxel intensities with higher pH (Figure 5E). This gives a quantitative indication that the silica material in the shell becomes denser when formed at higher pH. This is correlated to an inverse trend in the intensity of the core material that decreases with high pH until reaching the level of the hydrated surrounding (Figure 5E). Importantly, when we performed comparable electron tomography experiments on dried silica particles, the voxel intensities of particle shells that formed at the various pH values were similar (Figure S9). These differences between cryo and room temperature experiments indicate drying‐related modifications to the initial materials, which are substantial in the particles formed at the lower pH values, and suggest that the particles formed at pH 8 were originally more hydrated than the core‐shell particles that formed at pH 9.

To follow the process of hollow sphere formation at pH 9, we cryo‐fixed particles at different time points during the silicification process. The cryoET data collected from immature particles show a rapid enlargement of cavities on the expense of the shells within a few seconds of reaction initiation (Figure 5F), as quantified by measuring the volume fraction of the hollow cores that grow over time (Figure 5G). These observations suggest that condensate silicification at pH 9 starts from a homogenous particle into which soluble silicate is diffusing from the dilute phase. However, unlike the process in pH 8, silica condensation is not homogenous inside the particle but continuously enlarges the inner core.

A plausible explanation for this process can emerge from the fact that silicate species at pH 9 (Si(OH)_3_O^−^) are more negatively charged compared to the neutral silicic acid (Si(OH)_4_) that dominates at pH 8. The condensation of charged silicates involves the release of hydroxide ions, instead of only water molecules that are the products of lower pH condensation (Figure 5H). This additional base can either diffuse out of the particle or accumulate inside it. We propose that the inner core is the result of base accumulation in the particle core that creates a more basic environment, in which the silanol bonds are weaker and can undergo iterative dissolution and polymerization of the silica, which drives its gradual densification in the shell material (Figure 5H). To probe core formation and pH changes during silicification, a pH‐sensitive dye, HPTS, was introduced to the PAH droplets and monitored by live fluorescence microscopy. While direct visualization of an alkaline core was hindered by the strong affinity of HPTS for the polymer matrix, time‐lapse imaging confirmed the formation of a core during silicification at pH 9 (Figure S10). Additionally, quantitative fluorescence measurements revealed a clear increase in HPTS emission intensity upon silicification in a pH dependent manner, providing experimental evidence for base production and pH elevation within the polymer–silica droplets (Figure S10).

**FIGURE 6:**
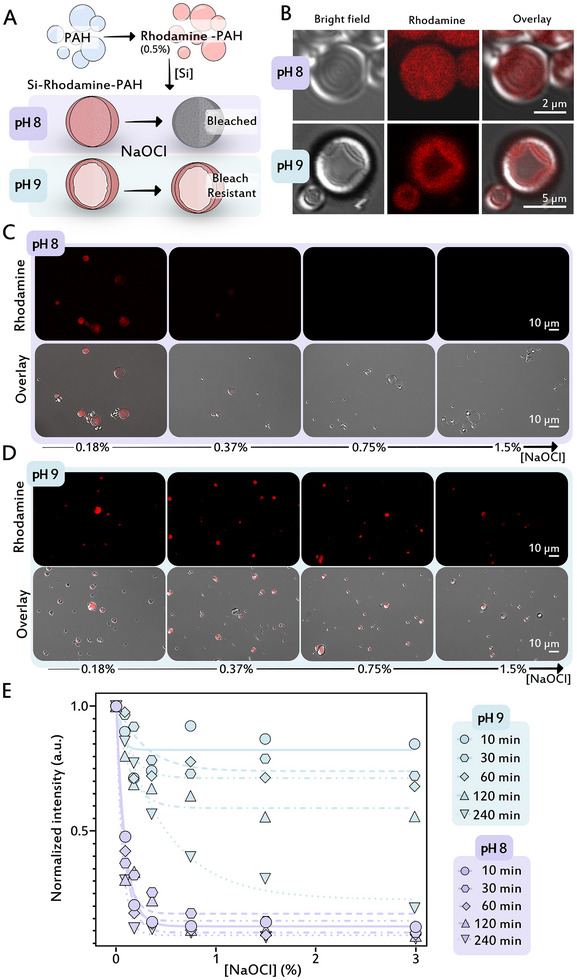
Variable solute accessibility in different silica particles. (A) Schematic illustration of the experimental plan. (B) Confocal fluorescence images of rhodamine‐tagged silica particles, formed at different pH values, show that the dye is integrated into the silica hybrid. (C,D) Confocal fluorescence and overlay images of silica particles after treatment with varying concentrations of NaOCl solution for 1 h. Rhodamine fluorescence bleaches faster in the particles that formed at pH 8. (E) Photoluminescence (PL) emission from particle suspensions exposed to different NaOCl concentrations over varying bleaching time shows a similar trend to the confocal images.

These observations suggest that changing silicification pH results in variable density and hydration level of particles. We performed a direct experiment to evaluate the accessibility of freely diffusing solutes to the hybrid silica. To this end, we expanded the synthesis protocol to add 0.5% of PAH that is covalently tagged with rhodamine (a fluorescent dye) to the PAH solution, which results in dyed condensates, leading to the formation of rhodamine‐tagged silica‐polymer hybrids after condensate silicification (Figure 6A,B). We exposed fluorescent particles to an oxidizing solution (NaOCl) and monitored the degradation of the dye by the bleach. In accordance with the previous results, the hydrated and uniform silica particles obtained from pH 8 exhibited rapid fluorescence bleaching, indicating high permeability and solute accessibility (Figure 6C). In contrast, the dense hollow silica particles that formed at pH 9 remained resistant to bleaching, suggesting restricted molecular transport and limited solvent exchange (Figure 6D,E). These observations demonstrate the more solvent‐accessible nature of the silica formed at pH 8, and open the option to use this tunable trait for functional materials.

## 3 Discussion

This work unfolds a chemical pathway for the synthesis of dense silica materials in aqueous environments, which might share similarities to the chemical conditions that underlie biological silicification reactions. This pathway hinges on the activity of polyamine condensates that act as chemical hotspots that facilitate the diffusion of soluble silicate species from the dilute phase into the condensate and support their subsequent polymerization. The simple conditions and regulators of this process, namely polyamine concentration and pH, are in marked difference to previously reported systems that used precise interactions between surfactants, catalysts, and silicate precursors to design new materials. The importance in using LLPS of the polyamine, in contrast to classical self‐assembly, protein aggregates, or micellar particles, is that the equilibrium state of the LLPS system can allow a general regulator like pH to tune independently the behavior of the templating system.

It was long known that polyamines are functional in biosilicification, even though the critical feature that makes them so important is unclear. The current work is highlighting macromolecular condensation as a general chemical regime that underlies their function as silicification templates. In addition, the reported involvement of active proton transport into the organelle of silica formation in diatoms can be rationalized in this context [50], as pH is shown here to be a regulator of the condensate silicification.

Importantly, this silicification strategy uses only biocompatible reactants in physiological conditions. In this initial work we have used a very generic polyamine, PAH, but the versatile toolbox of biomolecular condensates can be used to modulate and design the properties of the condensates [43, 45]. There is an immense body of knowledge on condensate formation, composition, and dynamics, which can be used to develop an arsenal of silica hybrids by modulating the structure and chemistry of the polyamines. Such materials can expand the established functionalities of nano‐ and meso‐porous silica that forms via the sol‐gel processes.

The results of the rhodamine‐labelled particles show the potential of controlling silica density with slight pH modification. This can be useful in biotechnology, where controlled drug release from mesoporous silica particles is highly investigated [7]. First, to incorporate a substrate into the silica particle, the condensate should be designed with specific chemical properties, such as covalent modifications, hydrophobicity, or affinity, that promote the preferential inclusion into the hybrid silica substrate. Second, the pH of the system, together with the silicate precursor and the nature of the polyamine, can serve to design the density and diffusivity within the hybrid materials. Therefore, the modular platform of condensates can mark the development of new silica materials.

An important aspect of this reaction is that it occurs outside of the ‘regular’ range of slightly acidic pH values, where silica is most insoluble. This is in accordance with the previous study of silicate and polyamine phase separation [39], and it offers great advantages as the reaction is going from one equilibrium state (an undersaturated precursor solution and phase separated droplets) to another (dense particles) and since the silicate in the dilute phase is undersaturated there is no competing process such as the formation of silica gel. It is important to note that using sodium silicate as the precursors, rather than alkoxysilane, means that there is no natural buffering to the system (which is the role of the basic catalysts in reactions such as the Stober method), opening new avenues for control over this system.

Lastly, the experiments at cryo conditions highlight important aspects about the mechanisms of the silicification process. It is clear that the preformed condensates are pivotal for silica polymerization, possibly explaining the silicophilic activity of the polyamines. We also show that dehydration can affect some properties of the forming silica, highlighting the importance of cryo and in situ techniques to study materials that form in aqueous conditions.

## 4 Conclusion

The ability to form dense silicified materials at aqueous conditions from soluble precursors is the hallmark of silicifying organisms. The current work demonstrates that polyamine condensates might be the functional handle to recapitulate this process in the lab. We show that phase separated polyamine condensates concentrate soluble silicate precursors from the surrounding dilute phase, and that these precursors polymerize in the condensate to form a solid hybrid material with compositional properties comparable to biosilica. The main chemical regulator that distinguishes this process from the unstructured formation of a silica gel is pH. Phase separation of the polyamine condensate and silica polymerization are pH‐dependent, occurring at higher pH values than gelation and allowing to control the composition and architecture of the silica particles. It will be of great importance to investigate how modifying the chemistry, rheology, and composition of the macromolecular condensates allows to design functional silica particles.

## Author Contributions

P.B. performed the experiments, collected and analyzed the data. N.L. and M.L. conducted and interpreted the solid‐state NMR experiments. L.A. isolated diatom silica from the culture. L.A., N.E. and R.K. assisted with tomography data acquisition and A.G. supervised the research. P.B. and A.G. wrote the manuscript with input from all authors.

## Funding

This research was funded by the European Union (ERC, LivingCrystals, 101169570).

## Conflicts of Interest

The authors declare no conflicts of interest.

## Supporting information




**Supporting File 1**: anie71563‐sup‐0001‐SuppMat.pdf.

**Supporting File 2**: anie71563‐sup‐0002‐MovieS1.mp4.

## Data Availability

The data that support the findings of this study are available in the Supporting Information of this article.
