# Supplementary material for: Macromolecular Condensates as Tunable Scaffolds for Bio‐Inspired Silica Hybrids
Source: Angew Chem Int Ed Engl. 2026 Feb 17;65(14):e22966. doi: 10.1002/anie.202522966 (PMC13023690; doi:10.1002/anie.202522966)
Supplement: Supplementary file 1 — Supporting File 1: anie71563‐sup‐0001‐SuppMat.pdf. [file ANIE-65-e22966-s001.pdf]

Supplementary information

Macromolecular Condensates as Tunable Scaffolds for Bio-inspired silica Hybrids

Protap Biswas[a], Lior Aram[a], Nitzan Livni[b], Roman Kamyshinsky[c], Nadav Elad[c], Michal Leskes[b], Assaf Gal[a]

[a]Department of Plant and Environmental Sciences, Weizmann Institute of Science, Rehovot 7610001, Israel
[b]Department of Molecular Chemistry and Materials Science, Weizmann Institute of Science, Rehovot 7610001, Israel
[c]Department of Chemical Research Support, Weizmann Institute of Science, Rehovot 7610001, Israel
*Correspondence to assaf.gal@weizmann.ac.il

Experimental Section

Materials and Methods**:** Polyallylamine hydrochloride (PAH·HCl, 17.5kDa and 50 kDa), Polyalylamine solution (20%, 65kDa) sodium silicate solution ($(NaOH)_x(Na_2SiO_3)_y \cdot zH_2O$, 27% SiO_2), Rhodamine B isothiocyanate, 8-Hydroxypyrene-1,3,6-trisulfonic acid trisodium salt and Sodium hypochloride solution (6%) were obtained from Sigma-Aldrich. Polyalylamine solution (20%, ~150kDa) were bought from Polysciences. All solutions were prepared using ultrapure water from a Milli-Q Ultrapure Lab Water System (Merck).

Synthesis: Phase separation of polymer droplets was achieved by adding NaOH to the hydrochloride salt of PAH·HCl. Stock solutions (100 mM) of polyallylamine (PAH) were prepared and diluted with Milli-Q water to the desired concentrations. NaOH (2.5 M) was used to adjust the pH of the polymer solution. The silicate/silicic acid stock solution was prepared by diluting the sodium silicate stock, and its pH was adjusted with concentrated HCl. As was previously shown,

these solutions are unreactive in the absence of the polymer.[39] For silicification, freshly prepared silicate solution and the polymer solution were mixed in equal volumes and left to silicify for 24 hours, except for silicification conditions below pH 8 (4 hours), as silicic acid auto-polymerizes due to its supersaturation index at low < pH 8. Silica particles were isolated by centrifugation (22,000 g, 5 min) and washed thrice with Milli-Q water. The samples were dried by lyophilization for dry-state analyses. For biosilica measurements, *Thalassiosira pseudonana* (CCMP1335) cultures were centrifuged, washed with MilliQ water, and treated with 0.06% sodium hypochlorite at 4 °C under agitation for ~8 hr. The solution was refreshed, and incubation continued for ~16 hr. Cells were then washed, lyophilized, and the silica used for dry-state studies.

Dynamic Light Scattering (DLS): Particle size distributions were analyzed using a Zetasizer Nano ZSP (Malvern Instruments, UK) equipped with a 633 nm laser. Measurements were recorded as intensity distributions, and average values were obtained from three replicates.

Polymer Titration: Titration experiments were conducted using a pH meter (Eutech pH 700). A PAH·HCl solution (25 mM, 10 mL) with an initial pH of 2 was titrated with NaOH (1 M) in 5 µL increments. pH changes were recorded, and titration points were determined from the first-order derivative of the titration curve. The first titration point was assumed to indicate the onset of deprotonation, while the endpoint was determined from the derivative curve of the polymer titration data. The degree of deprotonation and *pKa* values were calculated using previously reported methods.

Fourier Transform Infrared Spectroscopy (FT-IR): Dried samples were pressed into KBr plates using a hydraulic pelletizer, and IR spectra were recorded using a Nicolet Summit X FTIR Spectrometer (Thermo Scientific).

Thermogravimetric Analysis (TGA): Thermal analysis was conducted on dried samples using an SDT Q600 instrument (TA Instruments, USA). Samples were heated at a 10 K/min rate under an oxidative atmosphere with a constant airflow of 100 mL/min. Organic polymers were assumed to be fully combusted at 700 °C, allowing quantification of silica content.

Scanning Electron Microscopy (SEM): Dried samples were mounted on carbon tape attached to aluminum SEM stubs. A 4 nm iridium coating was applied using a CCU-010 compact coating unit

(Safematic). Images were acquired using a Zeiss Sigma 500 SEM equipped with an in-lens detector at an accelerating voltage of 4 kV (working distance: 2.5 mm).

Solid-State Nuclear Magnetic Resonance (NMR): NMR measurements were performed on a Bruker Avance Neo spectrometer (9.4 T) using a 4.0 mm double-resonance probe at room temperature with a magic angle spinning (MAS) rate of 12.5 kHz. The ^{29}Si MAS NMR spectra were acquired by direct excitation followed by high-power ^{1}H decoupling of 70 kHz during signal acquisition and a recycle delay of 300 s. The recycle delay was optimized to give fully relaxed spectra. ^{29}Si spectra were referenced to kaolinite (-92 ppm). Spectra were deconvoluted using DMfit and analyzed using MATLAB.

Transmission Electron Microscopy (TEM): The sample solution (2 µL) was drop-casted onto a carbon-coated copper TEM grid (200 mesh), and the excess solution was blotted off using filter paper. The grids were dried and stored in a vacuum desiccator before imaging. TEM images were acquired using an Tecnai T12 microscope (Thermo Fisher Scientific, USA) equipped with a TemCam-XF416 camera (TVIPS GmbH, Germany) at an accelerating voltage of 120 kV, as well as a Talos Arctica microscope (Thermo Fisher Scientific), as detailed below.

***Cryo-TEM and Tomography*:** Lacey carbon grids (Cu, 200 mesh) were plasma-cleaned, and 2 µL of the sample was applied to both sides of each grid. The grids were blotted for 2 seconds and vitrified in liquid ethane using an EM GP plunger (Leica Microsystems, Vienna, Austria) at 25 °C and 90% relative humidity. Vitrified samples were stored in liquid nitrogen prior to imaging. Cryo-TEM imaging was performed on a Talos Arctica microscope (Thermo Fisher Scientific) operated at 200 kV, using a Falcon 4i direct electron detector. Images were acquired at 45,000× magnification (corresponding to a pixel size of 0.31 nm). Cryo-electron tomography data were acquired on a Titan Krios G3i microscope (Thermo Fisher Scientific) operated at 300 kV, equipped with a Gatan K3 direct detector and a Gatan BioQuantum energy filter. Tomograms were recorded using a 100 µm objective aperture at a defocus of −3 µm, with a total electron dose of 97 $e^{-}/Å^2$ at 53,000× magnification (pixel size 0.19 nm). Tilt series were collected from −60° to +60° with 2° increments using the dose-symmetric scheme in Tomography 5 software (Thermo Fisher Scientific). Reconstruction of tomograms was performed using the IMOD software package with the patch-tracking alignment method, and 3D segmentation was carried out using Amira 3D software (version 2021.2, Thermo Fisher Scientific).

Synthesis of Rhodamine B-Tagged Silica Particles and Bleaching Assay: Rhodamine B isothiocyanate (RBITC; 1 mg/100 µL in ethanol) was used to label poly(allylamine hydrochloride) (PAH, MW ~50,000 Da). PAH (62.5 mg) was dissolved in 5 mL Milli-Q water, followed by the addition of 200 µL saturated $NaHCO_3$. The pH was adjusted to 7.4, and saturated NaCl was added to ensure full dissolution. RBITC stock (25 µL) was added, and the mixture was stirred overnight at room temperature in the dark. The reaction was dialyzed against Milli-Q water for 24 h (MWCO 14 kDa; Sigma-Aldrich), with three water changes. The labelled PAH was stored at 4 °C in the dark. Silicification was performed using PAH droplets prepared at 50 mM concentration, with 0.5 wt% Rhodamine-labeled PAH, at pH 8 and 9. After silica formation, 1 mL aliquots were treated with varying bleach concentrations. Confocal images were acquired from samples mounted on glass slides. For photoluminescence (PL) analysis, 200 µL samples were loaded into a 96-well plate and measured at λex/λem = 540/570 nm. PL intensities were normalized to untreated controls (0 min, 0% bleach) to assess bleaching resistance.

Confocal laser scanning fluorescence microscopy: The confocal images of the particles were acquired in Nikon A1 LFOV, the laser (561.5/595nm) was used for rhodamine-tagged particle imaging with 100X (silicon oil dispersion) or 60X (water dispersion) lenses. The images were processed with the built-in software NIS-Elements Viewer 5.22 of the microscope.

***Image and data analysis*:** Particle size and width measurements were performed using ImageJ software. The statistical size distribution of the particles from SEM images was determined by measuring the diameters of approximately 50 particles. The cavity size of hollow silica particles was calculated as a volume percentage by measuring the inner and outer diameters from TEM images. Voxel contrast analysis from tomographic slices was performed using 25 slices.

Gas adsorption study (BET): The N_2 adsorption/desorption isotherm of the silica particles was measured at 77 K after the samples were degassed at 200 °C under vacuum for 6h, using a Quantachrome Autosorb iQ2. The surface area was calculated by applying the Brunauer–Emmett–Teller (BET) model.

Supporting Figures

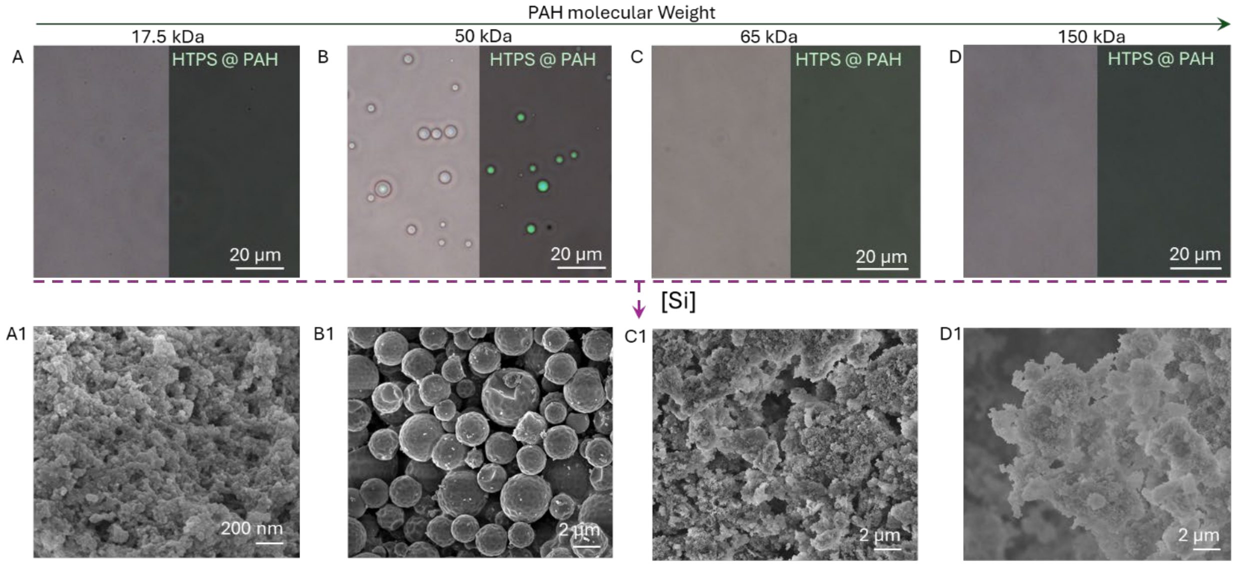

Figure S1. *Phase separation of PAH at various molecular lengths.* A-D) Optical images and corresponding overlay fluorescence images of the different molecular weight PAH solutions. The fluorescent dye HTPS stains forming condensates. The images show that for solutions at pH 9, phase separation is only evident for PAH 50kDa polymer. A1-D1) Corresponding SEM images of silica particles formed upon silicification using 50 mM silicate at pH9. A1, C1, D1) Soluble PAH (17.5 kDa, 65kDa, 150kDa) produced a random, loose network–like silica morphology; B1) Phase–separating PAH studied in this work (50 kDa) yields well-defined spherical silica particles templated by PAH polymer LLPS.

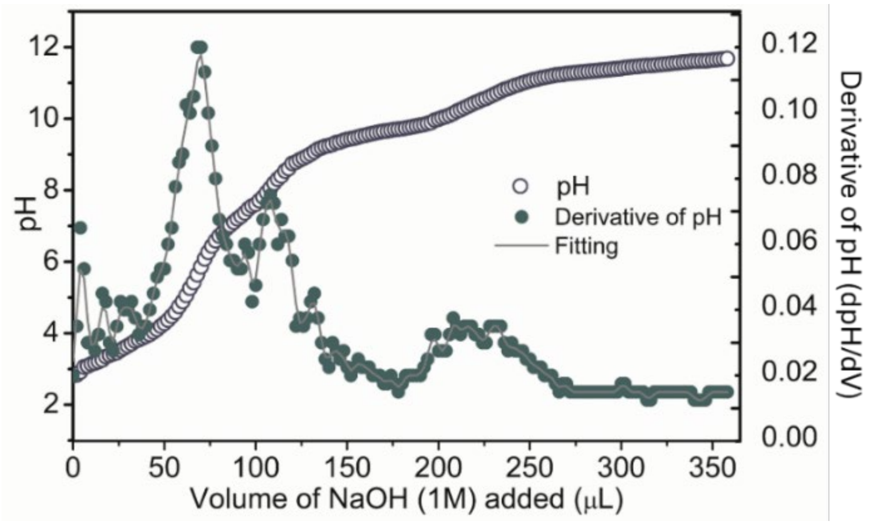

Figure S2. *pH-dependent deprotonation of PAH determined by titration.* The plot shows the change in pH upon gradual addition of NaOH to a PAH solution, illustrating the deprotonation behavior. The derivative curve (dpH/dV) is used to identify neutralization points, reflecting distinct deprotonation equilibria of the amine groups in PAH at different pH levels.

The data in Fig. 1 E was derived using the following calculations:

$[R\text{-}NH_3^+]$	+	$[OH^-]$	⇌	$[R\text{-}NH_2]$	
C_0		10^{-7}		0	Step 1: Dissociation is not allowed
$C_0 - x$		10^{-7}		x	Step 2: NaOH addition,<br>x = Concentration of NaOH added
$C_0 - x + y$		$10^{-7} + y$		$x - y$	Step 3: Allowing water dissociation, y.

$k_a = ([x - y]\,[10^{-7} + y]) / ([C_0 - x + y])$, $pk_a = -log\, k_a$

% Deprotonation = $\frac{[R-NH3+]}{[NaOH]}$ X 100

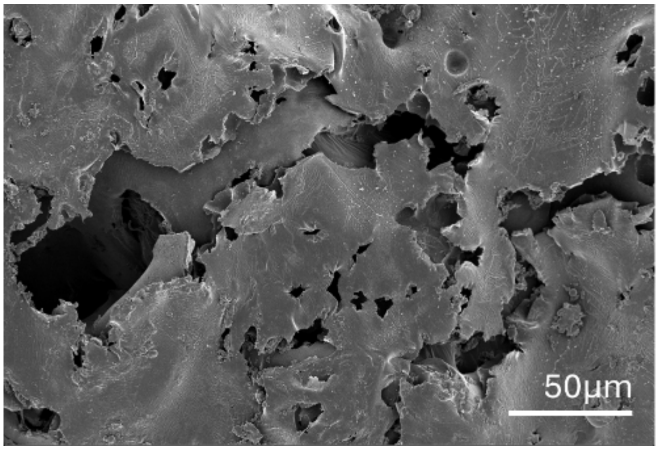

Figure S3: A representative SEM image of collapsed PAH droplets upon drying, which is strikingly different from the silicified droplets.

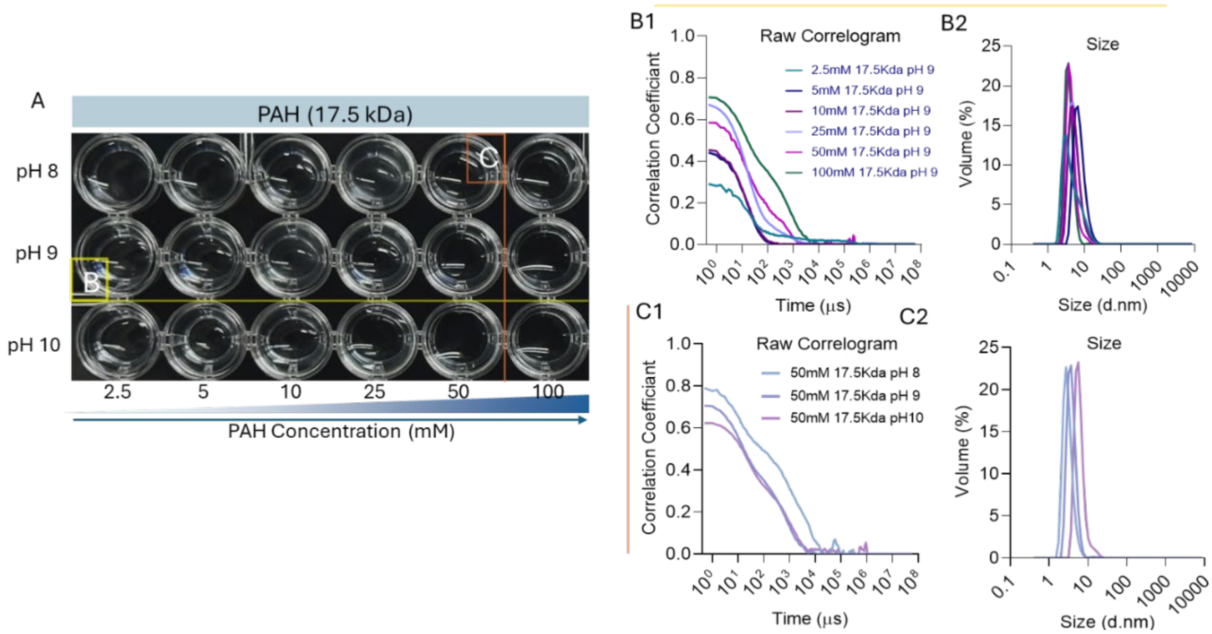

Figure S4. *Low-molecular-weight polyallylamine remains soluble across pH and concentration ranges.* PAH with a molecular weight of 17.5 kDa shows no phase separation under the tested conditions. Dynamic light scattering (DLS) measurements exhibit correlograms with a weak signal and small hydrodynamic radii, consistent with a soluble or non–phase-separated state of the individual polymer molecules. A) Optical images of PAH solutions across varying pH and concentration show no turbidity. B) DLS data of PAH solutions at pH 9 with concentrations ranging from 2.5–100 mM. C) DLS data of 50 mM PAH solutions at different pH values (pH 8–10).

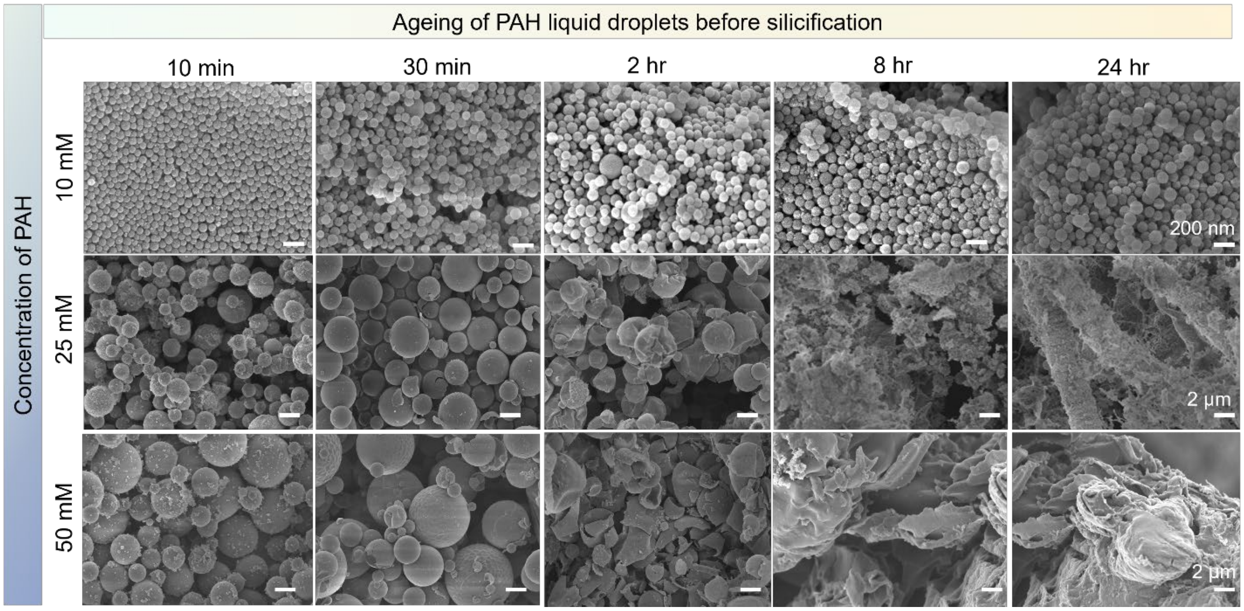

Figure S5. *Effect of aging and PAH concentration on size and morphology of the silica hybrids:* the fusion of LLPS droplets is accelerated at higher polyallylamine (PAH) concentrations and with increased aging time. Upon silicification, the resulting silica reflects the underlying droplet morphology. SEM images show that at low PAH concentration and short aging, discrete LLPS-templated silica particles form, while higher concentration and prolonged aging lead to coalesced silica precipitates.

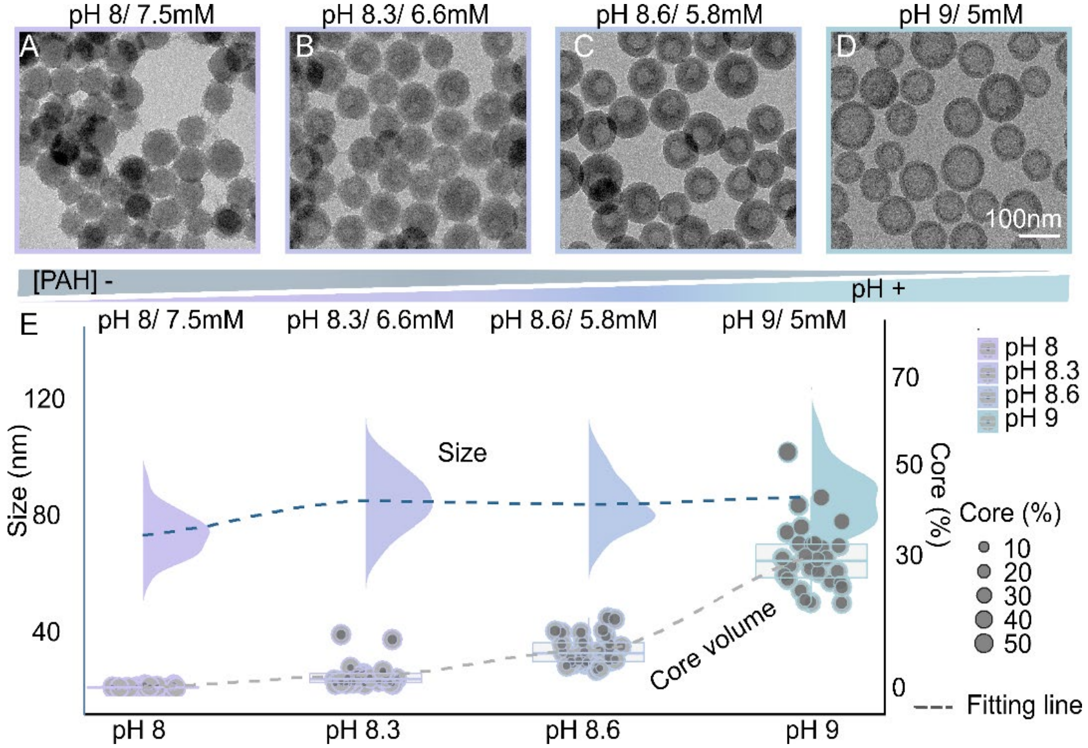

Figure S6: *Effect of PAH concentration and pH on silica particle size and core volume*. (A–D) TEM images of silica particles synthesized under different combinations of pH and PAH concentration. Conditions were selected to yield comparable overall particle sizes for a controlled comparison. (E) Particle size and core volume were quantified from the TEM images. Size distribution is presented using a hill plot, and core volume percentage is shown as a combination of scatter and box plots. The box represents 1.5× the interquartile range (IQR), and the central line indicates the mean core volume percentage.

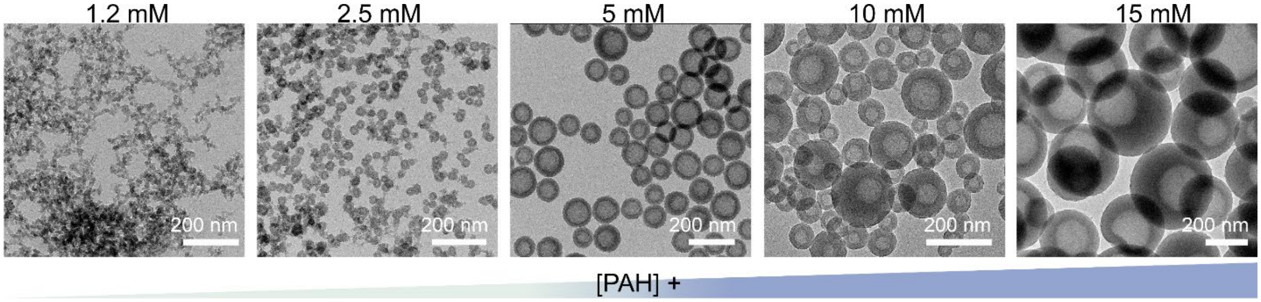

Figure S7. *Cavity formation in silica particles is governed by pH and is independent of particle size;* TEM images of silica particles synthesized at pH 9 with varying concentrations of PAH show that cavity formation occurs regardless of particle size.

A

Diatom		pH 8		pH 8.3		pH 8.6		pH 9	
Q	%	Q	%	Q	%	Q	%	Q	%
Q2	3.6 ± 0.2	Q2	1.29 ± 0.3	Q2	0.0 ± 0.2	Q2	2.51 ± 0.07	Q2	2.17 ± 0.07
Q3	33.1 ± 0.3	Q3	40.2 ± 0.2	Q3	52.5 ± 0.3	Q3	57.6 ± 0.2	Q3	64.7 ± 0.2
Q4	63.3 ± 0.3	Q4	58.5 ± 0.3	Q4	47.2 ± 0.3	Q4	39.8 ± 0.2	Q4	33.17 ± 0.2

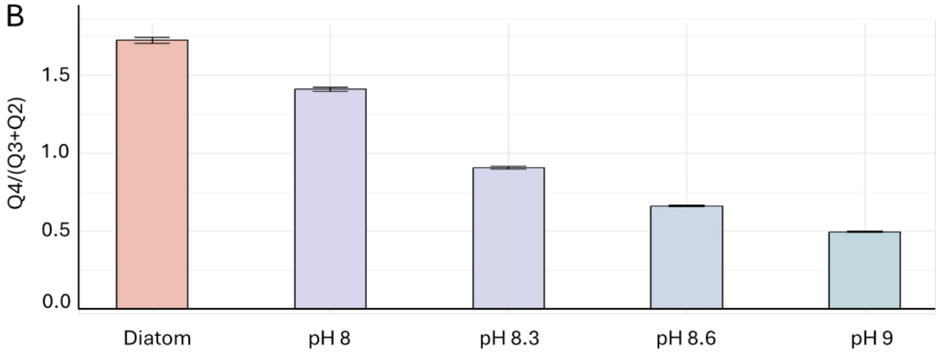

Figure S8: *[29] Si NMR analysis of silica samples across different pH conditions and comparison with diatom silica;* (A) Summary table of Q-peak intensities (Q^2, Q^3, and Q^4) measured by solid-state ^{29}Si NMR, for synthetic conditions at pH 8.0, 8.3, 8.6, 9.0 and isolated diatom silica, (B) Bar plot showing the relative extent of silica network polymerization, expressed by the arbitrary ratio $Q^4 / (Q^3 + Q^2)$, serves comparison for the degree of condensation among synthetic and biological samples. Error bars represent standard deviations.

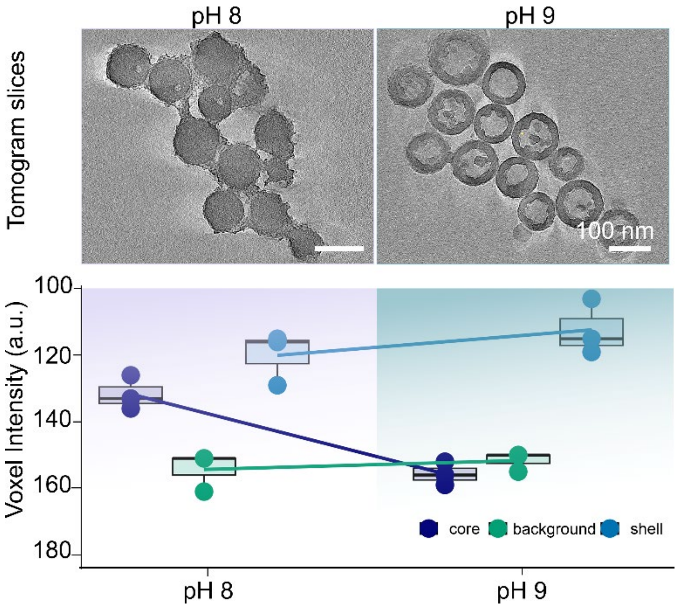

Figure S9: *Tomography of dried Si particles and voxel intensity comparison*: Voxel intensity analysis of tomographic datasets from different regions on the particles. Boxplots show median and IQR of mean voxel intensity; points are the individual sample means.

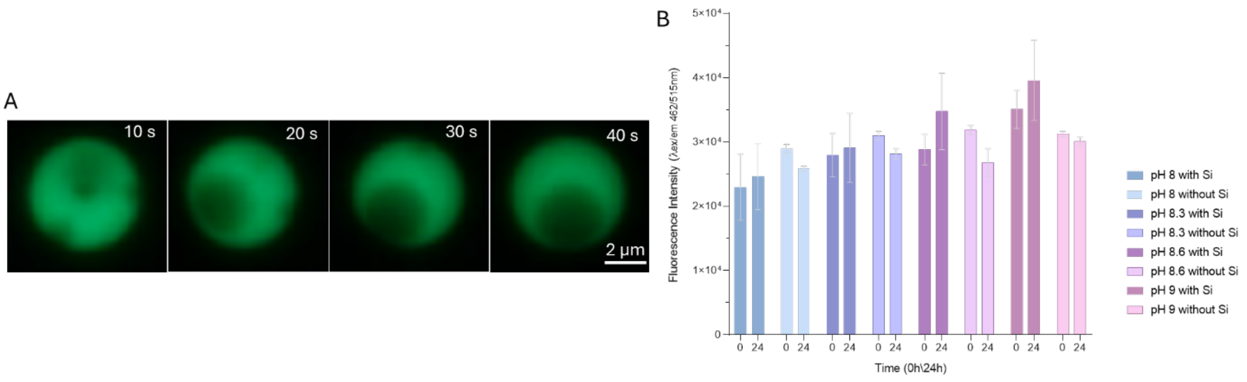

Figure S10: A) *Fluorescence microscopy of PAH droplets stained with HPTS during silicification at pH 9.* PAH droplets (50 mM, pH 9) were prepared in the presence of 8-hydroxypyrene-1,3,6-trisulfonic acid trisodium salt (HPTS, 10 µg), subsequently mixed with a 100 mM silicate solution at pH 9, and imaged by fluorescence microscopy to visualize core formation over time. Time-lapse fluorescence microscopy shows the evolution of the core during the silicification process (See also Movie S1). B) *Preparation and fluorescence characterization of HPTS-encapsulated PAH polymer droplets before and after silicification, showing enhanced emission intensity indicative of pH elevation.* Polymer droplets (total volume 10 mL) were prepared in the presence of HPTS under different pH/concentration conditions: pH 8.0 (10 mM), pH 8.3 (7.5 mM), pH 8.6 (6.5 mM), and pH 9.0 (5 mM). In all cases, 2.5 µg of HPTS (2.5 µL from a 1 mg mL^{-1} stock solution) was added per polymer solution. The HPTS-loaded droplets were silicified by addition of 100 mM silicic acid while maintaining the same pH as the corresponding polymer solution. The HPTS amount was standardized to 2.5 µg per 10 mL PAH solution, for which the dilute phase showed negligible fluorescence, as determined from the supernatant after centrifugation. Fluorescence emission intensities were measured using a plate reader (λex = 461 nm, λem = 515 nm) immediately after mixing (day 0) and after 24 h.

Table S1: The surface area measured from the BET isotherm from different samples upon lyophilization.

Sample	Surface area BET	
	set1 (m^2/g)	set2 (m^2/g)
pH 8	100.8	126
pH 8.3	100.5	89
pH 8.6	87	71
pH 9	105	77
Diatom	143.2	not measured
